# Risk Factors for Complications and Disease Recurrence after Ileocecal Resection for Crohn’s Disease in Children and Adults

**DOI:** 10.3390/biomedicines12040862

**Published:** 2024-04-13

**Authors:** Valeria Dipasquale, Erica Milone, Stefania Nigro, Angela Alibrandi, Enrica Antonelli, Donatella Di Fabrizio, Carmelo Romeo, Giuseppe Navarra, Claudio Romano

**Affiliations:** 1 Pediatric Gastroenterology and Cystic Fibrosis Unit, Department of Human Pathology in Adulthood and Childhood “G. Barresi”, University Hospital “G. Martino”, 98122 Messina, Italy; 2 Surgical Oncology Division, Department of Human Pathology in Adulthood and Childhood “G. Barresi”, University Hospital “G. Martino”, 98122 Messina, Italynigrostefania23@gmail.com (S.N.);; 3 Statistical and Mathematical Sciences Unit, Department of Economics, University of Messina, 98122 Messina, Italy; 4 Pediatric Surgery Unit, Department of Human Pathology in Adulthood and Childhood “G. Barresi”, University Hospital “G. Martino”, 98122 Messina, Italy; enri.antonelli@gmail.com (E.A.); dona.difabrizio@gmail.com (D.D.F.); romeoc@unime.it (C.R.)

**Keywords:** Crohn’s disease, disease recurrence, ileocecal resection, postoperative complications, risk factors, surgery

## Abstract

This study reports the complication and disease recurrence rates for ileocecal resection for pediatric and adult Crohn’s disease (CD) and identifies perioperative risk factors for these adverse outcomes in the two groups. Patients who underwent ileocecal resection for CD in a tertiary hospital in Italy (2010–2021) were included. Risk factors for postoperative complications and clinical and surgical disease recurrences were investigated with multivariate models. A total of 96 patients were included (children, 25%). There were no intraoperative complications. Thirty-one (32.3%) patients experienced 35 (36.5%) postoperative complications, and five (5.2%) were severe (Clavien–Dindo III–IV–V), with no intergroup difference for either overall postoperative complication rate (*p* = 0.257) or severe postoperative complication rate (*p* = 0.097). Most of these (77.1%) occurred within 30 days after surgery, especially in adults (*p* = 0.013). The multivariate analysis did not show risk factors for postoperative complications. Clinical and surgical recurrence rates after 5 years were 46.8% and 14.6%, respectively, with no intergroup rate differences. Clinical disease recurrence was positively correlated with previous abdominal surgery (*p* = 0.047) and negatively correlated with preoperative Hb levels (*p* = 0.046). A positive correlation was found between perianal disease and both clinical (*p* = 0.045) and surgical disease recurrences (*p* = 0.045). Urgent surgery was positively associated with surgical disease recurrence (*p* = 0.045). Notably, no children underwent urgent surgery in this study. In conclusion, the risk of postoperative complications among CD patients receiving ileocecal resection remains high, but most of them are nonserious. Some factors, such as urgent surgery, may increase the risk of disease recurrences.

## 1. Introduction

Crohn’s disease (CD) is a chronic inflammatory bowel disease (IBD) that can affect any segment of the gastrointestinal tract. Despite intensive medical treatment, long-standing refractory inflammation may cause irreversible damage to the bowel wall, resulting in stricturing or penetrating disease, which is best treated by surgical resection [1,2]. The risk of surgery after diagnosis is 16.3% at 1 year, 33.3% at 5 years, and 46.6% at 10 years, so about half of all patients undergo surgery in the 10 years after diagnosis [2,3]. Almost one-third of pediatric CD patients undergo surgery within 5 years of diagnosis [4,5,6], and the risk of having surgery is several times higher in children with a long-standing disease than in adults. The risk of surgery at the age of 30 for patients with the onset of CD in childhood is 48 ± 5% compared with 14 ± 2% for patients with adult-onset CD [7]. According to the LIR!C trial, laparoscopic resection can be considered a reasonable alternative to infliximab therapy in patients with limited, non-stricturing ileocecal Crohn’s disease in whom conventional therapy has failed [8]. Ileocecal resection is the most frequent surgical procedure in CD but exposes CD patients to a high risk of postoperative complications. Several risk factors for postoperative complications and/or disease recurrences following ileocecal resection have been identified in observational studies, but the majority of them failed to account for crucial confounding factors such as disease activity or previous or concurrent medication. Prior to surgery, it is critical to assess the patient’s specific risk in order to improve postoperative outcomes and reduce postoperative complications. Recurrence of postoperative disease is common; however, the risk can be reduced by the early start of immunomodulatory drug therapy [6]. The aims of this study were to describe the postoperative complication and disease recurrence rates following ileocecal resection in pediatric and adult CD and to identify possible risk factors for these adverse outcomes.

## 2. Materials and Methods

This is a retrospective cohort study of all CD patients who had an ileocecal resection at a tertiary referral center comprising both clinical and surgical IBD specialist units between January 2010 and December 2021. Crohn’s disease was diagnosed based on patient’s history and examination and supported by laboratory, serologic, radiologic, endoscopic, and histologic finings [9,10]. Primary ileocecal resection was defined as ileocecal resection as the first surgery for CD without prior intestinal resection, except for appendectomy. All procedures were performed by experienced pediatric surgeons.

### 2.1. Data Collection

Medical charts were reviewed for the following patient characteristics: gender, age at diagnosis, disease location according to the Paris classification, disease duration, smoking, age at surgery, preoperative or concomitant exposure to CD-related drugs, laboratory testing (hemoglobin, serum C-reactive protein, and albumin levels), preoperative enteral and/or parenteral nutrition, and postoperative therapy. The use of any steroids within 12 weeks of surgery was defined as preoperative steroid use. Preoperative anti-tumor necrosis factor alpha (anti-TNF-α) use was defined as patients using medication within 12 weeks before surgery. Surgical variables included type of surgical approach, type of anastomosis, additional surgical procedures, primary stoma rate, operating time, and the pathology report of the resected specimen.

### 2.2. Outcomes

Data on postoperative complications and disease recurrences were collected in a standardized format and reviewed in detail. A postoperative complication was defined as a surgical or other medical event after surgery. They were classified into four groups: (i) extra-abdominal infectious complications (including pneumonia and urinary tract infections); (ii) intra-abdominal septic complications (including wound infections, anastomotic leakage or anastomotic fistula, and intra-abdominal abscess); (iii) hemorrhagic complications; and (iv) other complications (including thromboembolic complications). The severity of each complication was graded according to the extent of the therapy that was necessary to resolve them according to the Clavien–Dindo classification [11]. Grades III–V were considered to be severe postoperative complications. Grade III requires surgical, endoscopic, or radiological intervention. Grade IV complications are life-threatening conditions and require intensive care/intensive care unit management. Grade V means the death of the patient. Clinical recurrence was defined as the reappearance of symptoms associated with objective signs of active disease after intestinal resection [12]. The pediatric Crohn’s Disease Activity Index (PCDAI) [13] and the Crohn’s Disease Activity Index (CDAI) [14] were used to assess the disease activity in children and adults, respectively. Surgical recurrence was defined as disease recurrence requiring new resection or strictureplasty for active inflammation or anastomotic strictures. Risk factors for postoperative complications and clinical and surgical disease recurrence were investigated.

### 2.3. Statistical Analysis

The numerical variables were described as mean and standard deviations (SD), and categorical variables as absolute frequency and percentages. The Chi Square test (or exact Fisher test or Likelihood ratio test, as appropriate) was applied to assess the existence of possible associations between categorical variables. A multivariable logistic regression model was estimated to identify significant predictors of post-operative complications (i.e., the explanatory power of the chosen covariates). The results were expressed as Odds Ratio (OR), 95% confidence interval (95% CI) and *p*-value. Statistical analyses were performed using IBM SPSS for Windows, Version 22 (Armonk, NY, USA, IBM Corp.). *p*-values lower than 0.05 were considered statistically significant.

## 3. Results

A total of 96 patients were included (mean age at surgery: 36.8 ± 18.2 years), of whom 25% (*n* = 24) were pediatric patients (0–17 years). Fifty-three (55.2%) patients were smokers, and they were all adults. Baseline characteristics of pediatric and adult patients are detailed in Table 1.

The mean follow-up period after surgery was 14.3 ± 18.1 years (7.67 ± 5.8 years and 10.2 ± 10.2 years for pediatric and adult patients, respectively). Resection specimens were all inflammation-free. Details about surgical procedures are presented in Table 2.

There were no intraoperative complications. Thirty-one (32.3%) patients experienced 35 (36.5%) postoperative complications, but only five (5.2%) were severe according to the Clavien–Dindo classification (Clavien–Dindo III–IV–V), with no intergroup difference for either overall postoperative complication rate (*p* = 0.257) or severe postoperative complication rate (*p* = 0.097). Postoperative complications were represented by intra-abdominal septic complications (anastomotic leakage, intra-abdominal collection, parietal abscess; *n* = 16, 16.7%; 7 children, 29.2%, vs. 9 adults, 12.5%, *p* = 0.058); extra-abdominal infections (*n* = 11, 11.5%; 4 children, 16.7%, vs. 7 adults, 9.7%, *p* = 0.355); hemorrhage (*n* = 4, 4.2%; 1 child, 4.2%, vs. 3 adults, 4.2%), thrombosis (*n* = 3, 3.1%, all adults), and death (*n* = 1, 1%, an adult patient). Most postoperative complications (*n* = 27, 77.1%) occurred within 30 days after surgery, especially in adults (*p* = 0.013). A minority of postoperative complications occurred immediately (*n* = 4, 4.2%) or after 30 days (*n* = 4, 4.2%). The management of severe postoperative complications was radiological (*n* = 2) or endoscopic (*n* = 3). The mean length of stay was 11.1 ± 6.1 days (11.9 ± 5.5 days and 10.9 ± 6.3 days for pediatric and adult patients, respectively).

The risk factors for postoperative complications are summarized in Table 3.

Extra-abdominal infections were the most frequent type of postoperative complication. The risk factors for intra-abdominal septic complications and extra-abdominal infections are summarized in Table S1. The presence of perianal disease was a risk factor for postoperative extra-abdominal infections (*p* < 0.001).

The clinical disease recurrence rate after 5 years was 46.8% (*n* = 45), while the surgical disease recurrence rate was 14.6% (*n* = 14). Thirty-eight (39.6%) patients started postoperative medical therapy (19 children, 79.2%, vs. 19 adults, 26.4%, *p* < 0.001), most of them (*n* = 23, 60.5%; 10 children, 52.6%, vs. 13 adults, 68.4%, *p* = 0.319) within 30 days after surgery. Postoperative therapy consisted of anti-TNF-α adalimumab.

Univariate analysis of risk factors for clinical and surgical disease recurrences is summarized in Table S2. Patients undergoing urgent surgery were most likely to develop a surgical disease recurrence (*p* = 0.029).

The multivariate analysis did not identify significant risk factors for postoperative complications among the following: pediatric vs. adult group, gender, age at diagnosis, age at surgery, disease location and duration, smoking, perianal disease, indication for surgery, timing, type of surgical access and anastomosis, preoperative exposure to drugs, preoperative laboratory values, preoperative enteral or parenteral nutrition, postoperative therapy, and disease recurrence (Table S3).

The multivariate linear regression model found that clinical disease recurrence was positively correlated with previous abdominal surgery (OR: 2.226, 95% CI: [1.624, 5.149], *p* = 0.047) and negatively correlated with preoperative Hb levels (OR: 0.797, 95% CI: [0.633, 0.903], *p* = 0.046). The positive correlation between clinical disease recurrence and previous abdominal surgery was confirmed by applying the multivariate linear regression model to the adult group only (OR: 2.200, 95% CI: [1.857, 5.645], *p* = 0.046). As presented in Table 1, previous abdominal surgery was significantly more frequent in the adult group than in the pediatric one (*p* = 0.002). A positive correlation was found between perianal disease and both clinical (OR: 8.225, 95% CI: [1.970, 9.719], *p* = 0.045) and surgical disease recurrences (OR: 4.145, 95% CI: [1.867, 19.822], *p* = 0.045). Urgent surgery was confirmed to be positively associated with surgical disease recurrence (OR: 5.250, 95% CI: [1.034, 26.656], *p* = 0.045). The multivariate analysis did not show any further significant risk factors for clinical and surgical disease recurrences (Tables S4 and S5).

## 4. Discussion

Despite recent advances in medical therapy, surgery remains an important component of CD treatment. In this retrospective study, we determined the postoperative complication and disease recurrence rates after ileocecal resection for CD and the predictors of these adverse outcomes in both adult and pediatric patients.

Indications for ileocecal resection in adult CD patients include stricture formation, fistula formation, perforation, failure of medical therapy (refractory disease), and cancer risk (in cases of long-standing inflammation in CD). Ileocecal resection may be indicated in pediatric CD patients for similar reasons as in adults, but there are some specific considerations for pediatric cases [15]. The decision to proceed with ileocecal resection should be made on an individual basis, considering factors such as disease severity, location and extent of involvement, the presence of complications, patient preferences, and overall health status. Like adults, pediatric patients with CD may not respond adequately to medical treatment, or they may experience intolerable side effects from medications. In such cases, surgery may be required to manage symptoms and complications [16]. Chronic inflammation and poor absorption of nutrients in Crohn’s disease can lead to growth impairment in children [16]. If medical therapy fails to promote growth or if the growth delay is severe, surgical resection of diseased bowel segments may be considered to improve nutritional status and promote growth. Other indications are strictures, fistulae and/or abscesses, and perforation. As with adults, the decision to proceed with ileocecal resection in pediatric CD patients should be made on a case-by-case basis, considering factors such as disease severity, extent of involvement, presence of complications, growth status, and patient preferences. Additionally, pediatric patients may require specialized care and multidisciplinary management involving pediatric gastroenterologists, surgeons, nutritionists, and other healthcare providers.

The literature reports rates of complications after ileocecal resection for CD that are quite variable, depending on different study designs, study sample characteristics, and disease phenotypes. In this study, the overall postoperative complication rate was 32.3%, which is consistent with previously reported percentages (21–37%) [17,18,19,20,21,22], with intra-abdominal septic complications being the most frequent. Moreover, in the present study, there was only one death in an adult, and severe postoperative complications (Clavien–Dindo III–IV–V) were observed in only 5.2% of patients, which was lower than in previous adult (11–13%) [20,21] and pediatric (10–27%) cohorts [6,23].

Notably, this is one of the few studies including both adult and pediatric CD patients. In the present study, no differences were observed in either the overall postoperative complication rate or the rate of severe postoperative complications. The only difference found between the two groups in terms of postoperative complication occurrence was that they were more likely to occur within 30 days of surgery in adults than in children (*p* = 0.013). In a nationwide prospective study performed by the REMIND group in nine French tertiary hospitals, postoperative complications occurred in 21% of 209 adult CD patients, with a severe complication rate of 9% [21]. In another adult cohort (*n* = 152) in the USA, an early postoperative complication rate of 37% was reported [18]. One patient died of sepsis (mortality rate: 0.7%) following the development of an enterocutaneous fistula with bile leakage [18]. In a retrospective cohort analysis of 122 pediatric CD patients who had ileocecal resection as their first intestinal resection for CD in seven tertiary centers in the Netherlands, the postoperative complication rate was 29.5.%, with a 10% severe postoperative complication rate [6]. Another pediatric study conducted by the EPIMAD group assessed the risk of postoperative complications in 128 children with CD in northern France and found out that 32 patients (25%) had at least one early (within 30 days of surgery) postoperative complication throughout the study period [19].

A variety of factors, including younger age at disease onset, more severe disease behavior, duration of disease prior to surgery, and inflammatory markers, have been documented to impact postoperative outcomes in adult CD patients [24,25]. However, none of these factors were associated with postoperative complications either in adults or children in the present study. In a study of 132 CD patients who had undergone surgical bowel resection, the presence of perianal disease independently increased the risk of reoperation (HR, 3.112; CI, 1.707–5.675) [26]. In this study, the absence of perianal disease was found to be a protective factor for postoperative extra-abdominal infections (*p* < 0.001). The cause of this relationship is unknown. A likely explanation is that perianal disease is associated with a more severe disease phenotype and represents one of the main indications for earlier, more aggressive therapy, which may lead to impaired immune system function and increased risk of infection in patients. The impact of medical treatment on postoperative complications has long been discussed. In contrast to previous studies [24], but in line with others [27], preoperative steroid exposure was not significantly associated with postoperative complications in this study. In a very recent retrospective study, 426 patients with CD who underwent abdominal surgery between 2001 and 2018 were divided into two groups [27]. Patients in the first group were receiving immunosuppressive medication at the time of surgery, whereas patients in the second group had never had pharmacological therapy for CD prior to surgery. There was no statistically significant difference in postoperative complications between the two groups [27]. The risk of postoperative complications associated with anti-TNF-α agents in CD remains debatable, with no clear consensus [21]. In the current study, neither preoperative nor postoperative anti-TNF-α drug use was associated with postoperative complications.

Clinical and surgical recurrence were observed in 46.8% and 14.6% of subjects, respectively, 5 years after resection, in line with previously reported rates. No intergroup differences either for clinical or surgical recurrence rates were observed in the present study. In some previous pediatric cohorts [6,28], surgical disease recurrence was observed in 17% and 11.9% of subjects at 5 years following intestinal resection, respectively.

Currently available evidence and guidelines recommend prophylactic medication (anti-TNF-α agents or thiopurines) after ileocecal resection to prevent endoscopic postoperative recurrence, especially in CD patients who are at high risk of postoperative recurrence or who have extensive disease [15,16,29]. A 2023 retrospective study concluded that the top-down strategy should be preferred to prevent short- and long-term postoperative recurrence in CD [30]. In this study, however, prophylaxis with anti-TNF-α agents was not protective against clinical or surgical disease recurrence. It is important to note that this study included patients who had surgery before the implementation of recommendations for starting postoperative prophylactic therapy in all patients with surgically induced remission, which might have biased the results. Notably, children were more likely to start postoperative adalimumab in comparison to adults (*p* < 0.001).

In the literature, numerous factors have been associated with disease recurrence after ileocecal resection for CD. Preliminary studies show that new surgical modalities like an antimesenteric functional end-to-end anastomosis (Kono-S) may decrease disease recurrence [31]. In this study, five patients had recently received a Kono-S anastomosis; therefore, it was not possible to explore the influence of this surgical method on the postoperative outcomes. In this cohort, previous abdominal surgery, low Hb levels, and the presence of perianal disease were found to be risk factors for clinical disease recurrences, while the presence of perianal disease and urgent surgery were risk factors for surgical disease recurrences. These factors may be landmarks of more severe disease phenotypes and/or medically refractory disease both in adult and pediatric CD, and this may explain the greater chance of recurrent disease after the ileocecal resection. A recent meta-analysis of postoperative outcomes in CD patients receiving urgent and elective intestinal resection identified 22 studies, with 955 patients undergoing urgent surgery and 6518 patients undergoing elective surgery. When compared to elective surgery, urgent surgery was associated with an increase in overall complications (RR = 1.43, 95% CI [1.09; 1.87], *p* = 0.010) and intraabdominal septic complications (RR = 1.44, 95% CI [1.08; 1.92], *p* = 0.013) [32]. Notably, at baseline, adults had undergone previous abdominal surgery more frequently than children (*p* = 0.002) and had significantly lower Hb levels in comparison to children (*p* = 0.002). Furthermore, no children underwent urgent surgery in this study.

This study has some limitations. First, the design was retrospective, so the included cases and clinical management approaches were the confounding variables. Furthermore, the retrospective study design may have limited clinical findings and management. Second, the sample size was small, making it difficult to make firm conclusions. Third, modifications in CD management occurred throughout the study period (for example, the introduction of anti-TNF-α drugs for pediatric CD). Fourth, many interesting variables have not been included, such as intestinal microbiota or genetics. A strength of this study is that it focused on ileocecal resection in a well-characterized, homogeneous population, which allowed analysis of factors associated with postoperative complications and disease recurrence, while controlling for important confounding variables such as disease activity and previous or concomitant therapy was taken into account. Another strength of this study is that it included both pediatric and adult CD patients and provided separate analyses for the two subgroups, although adult patients are more prevalent than pediatric patients. Despite its limitations, this study contributes to the body of evidence assessing the real burden of postoperative sequelae and disease recurrence after ileocecal resection for CD.

## 5. Conclusions

According to the current data, the likelihood of postoperative complications in CD patients receiving ileocecal resection remains significant and does not appear to have changed even after the use of biological drugs. However, the rate of severe postoperative complications is low. The absence of perianal disease may be protective against postoperative extra-abdominal infections. Furthermore, clinical disease recurrence may be more common in patients with previous abdominal surgery and lower baseline Hb levels, while surgical disease recurrence may be less common in those receiving elective surgery than in those undergoing urgent surgery. Perianal disease appears to be a risk factor for both clinical and surgical disease recurrences. Notably, children in the present cohort did not receive urgent surgery. Postoperative complications and disease recurrence are major concerns in the surgical management of both pediatric and adult CD patients. Identifying and anticipating predictors is critical to delaying or perhaps preventing these adverse outcomes, consistent with the precision medicine model and personalization of therapies in IBD. Future efforts could be directed toward reducing the rate of urgent surgery in adults.

## Figures and Tables

**Table 1:** Baseline characteristics of all included patients.

Variable	Children (*n* = 24)	Adults (*n* = 72)	*p*
Gender, *n* (%)			0.626
Male	16 (66.7)	44 (61.1)	
Female	8 (33.3)	28 (38.9)	
Disease duration, years, mean (SD)	5.7 (4.3)	10.1 (9.1)	0.112
Disease location, *n* (%) ^a,b^			**0.045**
L1	8 (33.3)	45 (62.5)	
L2	3 (12.5)	5 (6.9)	
L3	13 (54.2)	22 (30.6)	
Perianal disease, *n* (%) ^a,b^			1
No	22 (91.7)	66 (91.7)	
Yes	2 (8.3)	6 (8.3)	
Stricturing disease, *n* (%) ^c^			0.430
No	4 (17.4)	8 (11.1)	
Yes	19 (82.6)	64 (88.9)	
Penetrating disease, *n* (%) ^c^			0.617
No	17 (70.8)	47 (65.3)	
Yes	7 (29.2)	25 (34.7)	
Previous abdominal surgery, *n* (%) *			**0.002**
No	20 (87)	36 (50)	
Yes	3 (13)	36 (50)	
Steroids, *n* (%) **			0.131
No	22 (91.7)	56 (77.8)	
Yes	2 (8.3)	16 (22.2)	
Biologics, *n* (%)			**0.009**
No	16 (66.7)	26 (43.8)	
Yes	8 (33.3)	46 (63.9)	
Methotrexate, *n* (%)			0.605
No	20 (83.3)	63 (87.5)	
Yes	4 (16.7)	9 (12.5)	
Thiopurines, *n* (%)			0.359
No	19 (79.2)	50 (69.4)	
Yes	5 (20.8)	22 (30.6)	
Mesalamine, *n* (%)			**0.020**
No	23 (95.8)	53 (79.2)	
Yes	1 (4.2)	20 (20.8)	
Enteral nutrition, *n* (%)			**0.018**
No	21 (87.5)	71 (98.6)	
Yes	3 (12.5)	1 (1.4)	
Parenteral nutrition, *n* (%)			0.394
No	23 (95.8)	65 (90.3)	
Yes	1 (4.2)	7 (9.7)	
Laboratory values, mean (SD)			
Hb (mg/dL)	11.2 (1.3)	12.5 (1.9)	**0.002**
CRP (mg/dL)	3.1 (2.2)	2.6 (2.9)	0.418
Albumin (mg/dL)	40 (3.1)	36.5 (6.4)	0.325
Leukocytes (cells/μL)	10,113 (5223)	8055 (2211)	0.240

SD, standard deviation; Hb, hemoglobin; CRP, C-reactive protein; ^a^ According to the Paris classification (used to classify the severity of pediatric ulcerative colitis and Crohn’s disease based on specific categories); ^b^ According to the Montreal classification (used to classify the severity of ulcerative colitis and Crohn’s disease based on specific categories); ^c^ As indication for surgery; * ≥1 missing data; ** Some patients had more than one therapy.

**Table 2:** Surgical procedure data.

Variable	Children (*n* = 24)	Adults (*n* = 72)	*p*
Timing, *n* (%)			**0.040**
Elective	24 (100)	65 (90.3)	
Urgent	0	7 (9.7)	
Type of surgical access, *n* (%) *			0.207
Laparoscopy	18 (85.7)	52 (72.2)	
Laparotomy (open)	3 (14.3)	20 (27.8)	
Conversion (from laparoscopy to open), *n* (%) *			0.425
No	19 (95)	48 (88.9)	
Yes	1 (5)	6 (11.1)	
Type of anastomosis, *n* (%) *			0.944
Side-to-side	19 (86.4)	60 (83.3)	
End-to-side	2 (9.1)	8 (11.1)	
End-to-end ^a^	1 (4.5)	4 (5.6)	
Technique, *n* (%) *			0.769
Stapled	17 (85)	63 (87.5)	
Handsewn	3 (15)	9 (12.5)	
Additional procedures, *n* (%)			0.562
No	20 (83.3)	56 (77.8)	
Yes	4 (16.7)	16 (22.2)	
Postoperative drainage, *n* (%) *			**0.001**
No	3 (13.6)	0	
Yes	19 (86.4)	72 (100)	
Perioperative blood transfusion, *n* (%) *			0.462
No	20 (90.9)	61 (84.7)	
Yes	2 (9.1)	11 (15.3)	

^a^ Antimesenteric functional end-to-end anastomosis (Kono-S); * ≥1 missing data.

**Table 3:** Risk factors for postoperative complications after ileocecal resection for Crohn’s disease.

Variable	Postoperative Complications		*p*-Value
	No (*n* = 65)	Yes (*n* = 31)	
Group, *n* (%)			0.257
Pediatric	14 (21.5)	10 (32.3)	
Adult	51 (78.5)	21 (67.7)	
Gender, *n* (%)			0.778
Male	40 (61.5)	20 (64.5)	
Female	25 (38.5)	11 (35.5)	
Smoker, *n* (%)			0.172
No	26 (40)	17 (54.8)	
Yes	39 (60)	14 (45.2)	
Age at diagnosis, *n* (%) ^a^			0.171
A1a: <10 years	0	2 (6.5)	
A1b: 10–17 years	14 (21.5)	8 (25.8)	
A2: 17–40 years	36 (55.4)	15 (48.4)	
A3: >40 years	15 (23.1)	6 (19.4)	
Disease location, *n* (%) ^a,b^			0.288
L1	37 (56.9)	16 (51.6)	
L2	7 (10.8)	1 (3.2)	
L3	21 (32.3)	14 (45.2)	
Perianal disease, *n* (%) ^a,b^			0.263
No	61 (93.8)	27 (87.1)	
Yes	4 (6.2)	4 (12.9)	
Indications for ileocecal resection			
Stricturing disease, *n* (%) *			0.421
No	7 (10.8)	5 (16.7)	
Yes	58 (89.2)	25 (83.3)	
Penetrating disease, *n* (%)			0.537
No	42 (64.6)	22 (71)	
Yes	23 (35.4)	9 (29)	
Medications **			
Biologics, *n* (%)			0.847
No	28 (43.1)	14 (45.2)	
Yes	37 (56.9)	17 (54.8)	
Methotrexate, *n* (%)			0.445
No	55 (84.6)	28 (90.3)	
Yes	10 (15.4)	3 (9.7)	
Thiopurines, *n* (%)			0.891
No	47 (72.3)	22 (71)	
Yes	18 (27.7)	9 (29)	
Mesalamine, *n* (%)			0.805
No	51 (78.5)	25 (80.6)	
Yes	14 (21.5)	6 (19.4)	
Enteral nutrition, *n* (%)			0.750
No	62 (95.4)	30 (96.8)	
Yes	3 (4.6)	1 (3.2)	
Parenteral nutrition, *n* (%)			0.645
No	59 (90.8)	29 (93.5)	
Yes	6 (9.2)	2 (6.5)	
Surgical data			
Timing of surgery, *n* (%)			0.535
Elective	61 (93.8)	28 (90.3)	
Urgent	4 (6.2)	3 (9.7)	
Type of surgical access, *n* (%) *			0.214
Laparoscopy	45 (71.4)	25 (83.3)	
Laparotomy (open)	18 (28.6)	5 (16.7)	
Conversion (from laparoscopy to open), *n* (%) *			0.252
No	43 (87.8)	24 (96)	
Yes	6 (12.2)	1 (4)	
Type of anastomosis, *n* (%) *			0.730
Side-to-side	54 (84.4)	25 (83.3)	
End-to-side	6 (9.4)	4 (13.3)	
End-to-end	4 (6.2)	1 (3.3)	
Technique, *n* (%) *			0.547
Stapled	53 (85.5)	27 (90)	
Handsewn	9 (14.5)	3 (10)	
Additional procedures, *n* (%)			0.805
No	51 (78.5)	25 (80.6)	
Yes	14 (21.5)	6 (19.4)	
Postoperative drainage, *n* (%) *			0.228
No	3 (4.7)	0	
Yes	61 (95.3)	30 (100)	
Perioperative blood transfusion, *n* (%) *			0.522
No	57 (87.7)	24 (82.8)	
Yes	8 (12.3)	5 (17.2)	
Postoperative therapy, *n* (%)			0.223
No	42 (64.6)	16 (51.6)	
Yes	23 (35.4)	15 (48.4)	
Previous abdominal surgery, *n* (%) *			0.145
No	41 (64.1)	15 (48.4)	
Yes	23 (35.9)	16 (51.6)	
Disease recurrence			
Clinical, *n* (%) *			0.059
No	38 (59.4)	12 (38.7)	
Yes	26 (40.6)	19 (61.3)	
Surgical, *n* (%) *			0.726
No	54 (84.4)	27 (87.1)	
Yes	10 (15.6)	4 (12.9)	

^a^ According to the Paris classification (used to classify the severity of pediatric ulcerative colitis and Crohn’s disease based on specific categories); ^b^ According to the Montreal classification (used to classify the severity of ulcerative colitis and Crohn’s disease based on specific categories); * ≥1 missing data; ** Some patients had more than one therapy.

## Data Availability

The datasets generated during the current study are available from the corresponding author on reasonable request.

## Supplementary Materials

The following supporting information can be downloaded at: https://www.mdpi.com/article/10.3390/biomedicines12040862/s1, Table S1: Risk factors of intra-abdominal septic complications and extra-abdominal infections after ileocecal resection for Crohn’s disease; Table S2: Risk factors of disease recurrence after ileocecal resection for Crohn’s disease; Table S3: Results of multivariate logistic regression models for postoperative complications in Crohn’s disease patients; Table S4: Results of multivariate logistic regression models for clinical disease recurrence in Crohn’s disease patients; Table S5: Results of multivariate logistic regression models for surgical disease recurrence in Crohn’s disease patients.
