# Supplementary material for: Risk Factors for Complications and Disease Recurrence after Ileocecal Resection for Crohn’s Disease in Children and Adults
Source: Biomedicines. 2024 Apr 13;12(4):862. doi: 10.3390/biomedicines12040862 (PMC11047859; doi:10.3390/biomedicines12040862)
Supplement: Supplementary file 1 [file biomedicines-12-00862-s001.zip › Table S1_new.docx]

Table S1. Risk factors of intra-abdominal septic complications and extra-abdominal infections after ileocecal resection for Crohn’s disease

| Variable | Intra-abdominal septic complications | | p-value | Extra-abdominal infections | | p-value |
| --- | --- | --- | --- | --- | --- | --- |
|  | No (n = 80) | Yes (n = 16) |  | No (n = 85) | Yes (n = 11) |  |
| Group, n (%)  Pediatric  Adult | 17 (21.2)  63 (78.8) | 7 (43.8)  9 (56.2) | 0.058 | 20 (23.5)  65 (76.5) | 4 (36.4)  7 (63.6) | 0.355 |
| Gender, n (%)  Male  Female | 51 (63.8)  29 (36.2) | 9 (56.2)  7 (43.8) | 0.572 | 53 (62.4)  32 (37.6) | 7 (63.6)  4 (36.4) | 0.934 |
| Smoker, n (%)  No  Yes | 33 (41.2)  47 (58.8) | 10 (62.5)  6 (37.5) | 0.119 | 38 (44.7)  47 (55.3) | 5 (45.5)  6 (54.5) | 0.963 |
| Age at diagnosis, n (%) ^a^  A1a: < 10 years  A1b: 10 – 17 years  A2: 17 – 40 years  A3: > 40 years | 1 (1.2)  16 (20)  44 (55)  19 (23.8) | 1 (6.2)  6 (37.5)  7 (43.8)  2 (12.5) | 0.215 | 1 (1.2)  19 (22.4)  45 (52.9)  20 (23.5) | 1 (9.1)  3 (27.3)  6 (54.5)  1 (9.1) | 0.265 |
| Disease location, n (%) ^a, b^  L1  L2  L3 | 48 (60)  7 (8.8)  25 (31.2) | 5 (31.2)  1 (6.2)  10 (62.5) | 0.059 | 46 (54.1)  8 (9.4)  31 (36.5) | 7 (63.6)  0  4 (36.4) | 0.350 |
| Perianal disease, n (%) ^a, b^  No  Yes | 72 (90)  8 (10) | 16 (100)  0 | 0.080 | 81 (95.3)  4 (4.7) | 7 (63.6)  4 (36.4) | **<0.001** |
| Indications for ileocecal resection | | | |  | | |
| Stricturing disease, n (%) *  No  Yes | 9 (11.4)  70 (88.6) | 3 (18.8)  13 (81.2) | 0.419 | 10 (11.9)  74 (88.1) | 2 (18.2)  9 (81.8) | 0.556 |
| Penetrating disease, n (%)  No  Yes | 53 (66.2)  27 (33.8) | 11 (68.8)  5 (31.2) | 0.846 | 57 (67.1)  28 (32.9) | 7 (63.6)  4 (36.4) | 0.821 |
| Medications ** | | | |  | | |
| Steroids, n (%)  No  Yes | 63 (78.8)  17 (21.2) | 15 (93.8)  1 (6.2) | 0.161 | 69 (81.2)  16 (18.8) | 9 (81.8)  2 (18.2) | 0.959 |
| Biologics, n (%)  No  Yes | 33 (41.2)  47 (58.8) | 9 (56.2)  7 (43.8) | 0.270 | 37 (43.5)  48 (56.5) | 5 (45.5)  6 (54.5) | 0.904 |
| Methotrexate, n (%)  No  Yes | 69 (86.2)  10 (15.4) | 28 (90.3)  3 (9.7) | 0.894 | 73 (85.9)  12 (14.1) | 10 (90.9)  1 (9.1) | 0.647 |
| Thiopurines, n (%)  No  Yes | 57 (71.2)  23 (28.8) | 12 (75)  4 (25) | 0.761 | 62 (72.9)  23 827.1) | 7 (63.6)  4 (36.4) | 0.518 |
| Mesalamine, n (%)  No  Yes | 62 (77.5)  18 (22.5) | 14 (87.5)  2 (12.5) | 0.369 | 67 (78.8)  18 (21.2) | 9 (81.8)  2 (18.2) | 0.818 |
| Enteral nutrition, n (%)  No  Yes | 76 (95)  4 (5) | 16 (100)  0 | 0.222 | 82 (96.5)  3 (3.5) | 10 (90.9)  1 (9.1) | 0.385 |
| Parenteral nutrition, n (%)  No  Yes | 73 (91.2)  7 (8.8) | 15 (93.8)  1 (6.2) | 0.741 | 78 (91.8)  7 (8.2) | 10 (90.9)  1 (9.1) | 0.923 |
| Surgical data | | | | | | |
| Timing of surgery, n (%)  Elective  Urgency | 75 (93.8)  5 (6.2) | 14 (87.5)  2 (12.5) | 0.380 | 78 (91.8)  7 (8.2) | 11 (100)  0 | 0.183 |
| Type of surgical access, n (%) *  Laparoscopy  Laparotomy (open) | 21 (27.3)  56 (72.7) | 2 (12.5)  14 (87.5) | 0.213 | 20 (24.4)  62 (75.6) | 3 (27.3)  8 (72.7) | 0.835 |
| Conversion (from laparoscopy to open), n (%) *  No  Yes | 53 (89.8)  6 (10.2) | 14 (93.3)  1 (6.7) | 0.679 | 60 (89.6)  7 (10.4) | 7 (100)  0 | 0.226 |
| Type of anastomosis, n (%) *  Side-to-side  End-to-side  End-to-end | 67 (85.9)  7 (9)  4 (5.1) | 12 (75)  3 (18.8)  1 (6.2) | 0.494 | 69 (83.1)  9 (10.8)  5 (6) | 10 (90.9)  1 (9.1)  0 | 0.511 |
| Technique, n (%) *  Stapled  Handsewn | 65 (85.5)  11 (14.5) | 15 (93.8)  1 (6.2) | 0.375 | 70 (86.4)  11 (13.6) | 10 (90.9)  1 (9.1) | 0.678 |
| Additional procedures, n (%)  No  Yes | 64 (80)  16 (20) | 12 (75)  4 (25) | 0.653 | 66 (77.6)  19 (22.4) | 10 (90.9)  1 (9.1) | 0.308 |
| Postoperative drainage, n (%) *  No  Yes | 3 (3.8)  75 (96.2) | 0  16 (100) | 0.286 | 3 (3.6)  80 (96.4) | 0  11 (100) | 0.383 |
| Perioperative blood transfusion, n (%) *  No  Yes | 68 (85)  12 (15) | 13 (92.9)  1 (7.1) | 0.432 | 73 (86.9)  11 (13.1) | 8 (80)  2 (20) | 0.550 |
| Postoperative therapy, n (%)  No  Yes | 51 (63.8)  29 (36.2) | 7 (43.8)  9 (56.2) | 0.135 | 54 (63.5)  31 (36.5) | 4 (36.4)  7 (63.6) | 0.083 |
| Previous abdominal surgery, n (%) *  No  Yes | 49 (62)  30 (38) | 7 (43.8)  9 (56.2) | 0.175 | 50 (59.4)  34 (40.5) | 6 (54.5)  5 (45.5) | 0.752 |
| Disease recurrence | | | | | | |
| Clinical, n (%) *  No  Yes | 44 (55.7)  35 (44.3) | 6 (37.5)  10 (62.5) | 0.184 | 46 (54.8)  38 (45.2) | 4 (36.4)  7 (63.6) | 0.250 |
| Surgical, n (%) *  No  Yes | 67 (84.8)  12 (15.2) | 14 (87.5)  2 (12.5) | 0.782 | 72 (85.7)  12 (14.3) | 9 (81.8)  2 (18.2) | 0.732 |

^a^ According to the Paris classification (used to classify the severity of pediatric ulcerative colitis and Crohn disease based on specific categories)

^b^ According to the Montreal classification (used to classify the severity of ulcerative colitis and Crohn disease based on specific categories)

* ≥ 1 missing data

** Some patients had more than one therapy
