# Supplementary material for: Risk Factors for Complications and Disease Recurrence after Ileocecal Resection for Crohn’s Disease in Children and Adults
Source: Biomedicines. 2024 Apr 13;12(4):862. doi: 10.3390/biomedicines12040862 (PMC11047859; doi:10.3390/biomedicines12040862)
Supplement: Supplementary file 1 [file biomedicines-12-00862-s001.zip › Table S2_new.docx]

Table S2. Risk factors of disease recurrence after ileocecal resection for Crohn’s disease

| Variable | Clinical disease recurrence | | p-value | Surgical disease recurrence | | p-value |
| --- | --- | --- | --- | --- | --- | --- |
|  | No (n = 50) | Yes (n = 45) |  | No (n = 81) | Yes (n = 14) |  |
| Group, n (%)  Pediatric  Adult | 14 (28)  36 (72) | 9 (20)  36 (80) | 0.363 | 21 (25.9)  60 (74.1) | 2 (14.3)  12 (85.7) | 0.348 |
| Gender, n (%)  Male  Female | 30 (60)  20 (40) | 30 (66.7)  15 (33.3) | 0.501 | 51 (63)  30 (37) | 9 (64.3)  5 (35.7) | 0.925 |
| Smoker, n (%)  No  Yes | 26 (52)  24 (48) | 16 (35.6)  29 (64.4) | 0.107 | 39 (48.1)  42 (51.9) | 3 (21.4)  11 (78.6) | 0.063 |
| Age at diagnosis, n (%) ^a^  A1a: < 10 years  A1b: 10 – 17 years  A2: 17 – 40 years  A3: > 40 years | 0  14 (28)  22 (44)  14 (28) | 2 (4.4)  7 (15.6)  29 (64.4)  7 (15.6) | 0.061 | 2 (2.5)  19 (23.5)  41 (50.6)  19 (23.5) | 0  2 (14.3)  10 (71.4)  2 (14.3) | 0.468 |
| Disease location, n (%) ^a, b^  L1  L2  L3 | 23 (46)  6 (12)  21 (42) | 29 (64.4)  2 (4.4)  14 (31.1) | 0.147 | 42 (51.9)  8 (9.9)  31 (38.3) | 11 (78.6)  0  3 (21.4) | 0.081 |
| Perianal disease, n (%) ^a, b^  No  Yes | 48 (96)  2 (4) | 39 (86.7)  6 (13.3) | 0.102 | 76 (93.8)  5 (6.2) | 11 (78.6)  3 (21.4) | 0.058 |
| Indications for ileocecal resection | | | |  |  |  |
| Stricturing disease, n (%)  No  Yes | 6 (12)  44 (88) | 5 (11.4)  39 (88.6) | 0.924 | 10 (12.5)  70 (87.5) | 1 (7.1)  13 (92.9) | 0.565 |
| Penetrating disease, n (%)  No  Yes | 35 (70)  15 (30) | 29 (64.4)  16 (35.6) | 0.564 | 56 (69.1)  25 (30.9) | 8 (57.1)  6 (42.9) | 0.377 |
| Medications * | | | |  |  |  |
| Steroids, n (%)  No  Yes | 40 (80)  10 (20) | 37 (82.2)  8 (17.8) | 0.783 | 64 (79)  17 (21) | 13 (92.9)  1 (7.1) | 0.222 |
| Biologics, n (%)  No  Yes | 23 (46)  27 (54) | 18 (40)  27 (60) | 0.555 | 38 (46.9)  43 (53.1) | 3 (21.4)  11 (78.6) | 0.075 |
| Methotrexate, n (%)  No  Yes | 44 (88)  6 (12) | 38 (84.4)  7 (15.6) | 0.615 | 70 (86.4)  11 (13.6) | 12 (85.7)  2 (14.3) | 0.943 |
| Thiopurines, n (%)  No  Yes | 39 (78)  11 (22) | 29 (64.4)  16 (35.6) | 0.144 | 60 (74.1)  21 (25.9) | 8 (57.1)  6 (42.9) | 0.195 |
| Mesalamine, n (%)  No  Yes | 38 (76)  12 (24) | 37 (82.2)  8 (17.8) | 0.458 | 63 (77.8)  18 (22.2) | 12 (85.7)  2 (14.3) | 0.501 |
| Enteral nutrition, n (%)  No  Yes | 49 (98)  1 (2) | 43 (95.6)  2 (4.4) | 0.496 | 78 (96.3)  3 (3.7) | 13 (92.9)  1 (7.1) | 0.554 |
| Parenteral nutrition, n (%)  No  Yes | 47 (94)  3 (6) | 40 (88.9)  5 (11.1) | 0.370 | 76 (93.8)  5 (6.2) | 11 (78.6)  3 (21.4) | 0.058 |
| Surgical data | | | | | | |
| Timing of surgery, n (%)  Elective  Urgency | 48 (96)  2 (4) | 40 (88.9)  5 (11.1) | 0.185 | 77 (95.1)  4 (4.9) | 11 (78.6)  3 (21.4) | **0.029** |
| Type of surgical access, n (%)  Laparoscopy  Laparotomy (open) | 10 (20.8)  38 (79.2) | 12 (27.3)  32 (72.7) | 0.469 | 17 (21.8)  61 (78.2) | 5 (35.7)  9 (64.3) | 0.261 |
| Conversion (from laparoscopy to open), n (%)  No  Yes | 37 (94.9)  2 (5.1) | 29 (85.3)  5 (14.7) | 0.166 | 59 (92.2)  5 (7.8) | 7 (77.8)  2 (22.2) | 0.169 |
| Type of anastomosis, n (%)  Side-to-side  End-to-side  End-to-end | 42 (85.7)  5 (10.2)  2 (4.1) | 37 (84.1)  5 (11.4)  2 (4.5) | 0.976 | 68 (86.1)  7 (8.9)  4 (5.1) | 10 (71.4)  3 (21.4)  1 (7.1) | 0.342 |
| Technique, n (%)  Stapled  Handsewn | 39 (83)  8 (17) | 41 (93.2)  3 (6.8) | 0.136 | 67 (85.9)  11 (14.1) | 13 (92.9)  1 (7.1) | 0.476 |
| Additional procedures, n (%)  No  Yes | 51 (78.5)  14 (21.5) | 25 (80.6)  6 (19.4) | 0.805 | 65 (80.2)  16 (19.8) | 10 (71.4)  4 (28.6) | 0.455 |
| Postoperative drainage, n (%)  No  Yes | 2 (4.1)  47 (95.9) | 0  44 (100) | 0.107 | 2 (2.5)  77 (97.5) | 0  14 (100) | 0.416 |
| Perioperative blood transfusion, n (%)  No  Yes | 43 (89.6)  5 (10.4) | 37 (82.2)  8 (17.8) | 0.306 | 69 (87.3)  10 (12.7) | 11 (78.6)  3 (21.4) | 0.383 |
| Previous abdominal surgery, n (%)  No  Yes | 34 (68)  16 (32) | 22 (48.9)  23 (51.1) | 0.059 | 48 (60)  32 (40) | 7 (50)  7 (50) | 0.484 |
| Postoperative therapy, n (%)  No  Yes | 33 (66)  17 (34) | 25 (55.6)  20 (44.4) | 0.297 | 49 (60.5)  32 (39.5) | 9 (64.3)  5 (35.7) | 0.788 |
| Postoperative anti-TNF-α, n (%)  No  Yes | 40 (81.6)  9 (18.4) | 34 (75.6)  11 (24.4) | 0.472 | 66 (81.5)  15 (18.5) | 9 (64.3)  5 (35.7) | 0.145 |

TNF-α, tumor necrosis factor alpha

^a^ According to the Paris classification (used to classify the severity of pediatric ulcerative colitis and Crohn disease based on specific categories)

^b^ According to the Montreal classification (used to classify the severity of ulcerative colitis and Crohn’s disease based on specific categories)

* Some patients had more than one therapy
