# Supplementary material for: Risk Factors for Complications and Disease Recurrence after Ileocecal Resection for Crohn’s Disease in Children and Adults
Source: Biomedicines. 2024 Apr 13;12(4):862. doi: 10.3390/biomedicines12040862 (PMC11047859; doi:10.3390/biomedicines12040862)
Supplement: Supplementary file 1 [file biomedicines-12-00862-s001.zip › Table S3_new.docx]

Table S3. Results of multivariate logistic regression models for postoperative complications in Crohn’s disease patients

|  |  | **Multivariate** |  |
| --- | --- | --- | --- |
| **Variable** | **OR** | **[95% CI]** | ***p*-value** |
| Group (pediatric vs adult) | 0.576 | [0.221, 1.502] | 0.260 |
| Gender (male vs female) | 0.880 | [0.362, 2.142] | 0.778 |
| Age at diagnosis | 0.672 | [0.370, 1.221] | 0.192 |
| Age at surgery | 0.998 | [0.975, 1.022] | 0.889 |
| Smoker | 0.549 | [0.231, 1.303] | 0.174 |
| Disease duration | 1.001 | [0.996, 1.005] | 0.791 |
| Disease location (ileal) | 0.649 | [0.265, 1.588] | 0.343 |
| Disease location (ileocolonic) | 0.214 | [0.024, 1.937] | 0.170 |
| Perianal disease | 2.259 | [0.526, 9.710] | 0.273 |
| Stricturing disease | 0.603 | [0.175, 2.085] | 0.425 |
| Penetrating disease | 0.747 | [0.296, 1.888] | 0.538 |
| Steroids (preop. therapy) | 0.769 | [0.248, 2.390] | 0.650 |
| Biologics (preop. therapy) | 0.919 | [0.388, 2.174] | 0.847 |
| Methotrexate (preop. therapy) | 0.589 | [0.150, 2.315] | 0.449 |
| Thiopurines (preop. therapy) | 1.068 | [0.414, 2.753] | 0.891 |
| Mesalazine (preop. therapy) | 0.874 | [0.300, 2.547] | 0.806 |
| Enteral nutrition | 0.689 | [0.069, 6.904] | 0.751 |
| Parenteral nutrition | 0.678 | [0.129, 3.570] | 0.647 |
| Hemoglobin | 0.877 | [0.691, 1.112] | 0.278 |
| C-reactive protein | 0.852 | [0.558, 1.301] | 0.459 |
| Albumin | 1.050 | [0.781, 1.411] | 0.747 |
| Leukocytes | 1.000 | [1.000, 1.000] | 0.979 |
| Previous abdominal surgery | 1.901 | [0.797, 4.539] | 0.148 |
| Timing (elective vs emergency) | 1.634 | [0.343, 7.794] | 0.538 |
| Type of surgical access | 2.000 | [0.663, 6.038] | 0.219 |
| Conversion | 0.299 | [0.034, 0.299] | 0.276 |
| Type of anastomosis | 1.072 | [0.461, 2.491] | 0.871 |
| Technique | 1.528 | [0.382, 6.114] | 0.549 |
| Additional procedures | 0.874 | [0.300, 2.547] | 0.806 |
| Blood transfusion | 1.484 | [0.440, 5.002] | 0.524 |
| Length of stay | 1.023 | [0.956, 1.095] | 0.505 |
| Postoperative therapy | 1.712 | [0.718, 4.081] | 0.225 |
| Timing of postoperative therapy | 0.650 | [0.168, 2.516] | 0.533 |
| Disease recurrence | 2.314 | [0.962, 5.569] | 0.061 |
| Clinical disease recurrence | 2.036 | [0.848, 4.885] | 0.111 |
| Surgical disease recurrence | 0.800 | [0.230, 2.787] | 0.726 |
