# Supplementary material for: Risk Factors for Complications and Disease Recurrence after Ileocecal Resection for Crohn’s Disease in Children and Adults
Source: Biomedicines. 2024 Apr 13;12(4):862. doi: 10.3390/biomedicines12040862 (PMC11047859; doi:10.3390/biomedicines12040862)
Supplement: Supplementary file 1 [file biomedicines-12-00862-s001.zip › Table S4_new.docx]

Table S4. Results of multivariate logistic regression models for clinical disease recurrence in Crohn’s disease patients

|  |  | **Multivariate** |  |
| --- | --- | --- | --- |
| **Variable** | **OR** | **[95% CI]** | ***p*-value** |
| Group (pediatric vs adult) | 1.374 | [0.534, 3.536] | 0.510 |
| Gender (male vs female) | 0.656 | [0.283, 1.520] | 0.326 |
| Age at diagnosis | 0.783 | [0.447, 1.373] | 0.394 |
| Age at surgery | 0.998 | [0.976, 1.021] | 0.887 |
| Smoker | 1.611 | [0.712, 3.644] | 0.252 |
| Disease duration | 1.004 | [0.999, 1.009] | 0.109 |
| Disease location | 0.841 | [0.546, 1.294] | 0.431 |
| Perianal disease | 8.225 | [1.970, 9.719] | **0.045** |
| Stricturing disease | 0.775 | [0.219, 2.740] | 0.693 |
| Penetrating disease | 1.671 | [0.702, 3.976] | 0.246 |
| Steroids (preop. therapy) | 1.351 | [0.482, 3.791] | 0.567 |
| Biologics (preop. therapy) | 1.050 | [0.466, 2.365] | 0.906 |
| Methotrexate (preop. therapy) | 1.225 | [0.379, 3.960] | 0.735 |
| Thiopurines (preop. therapy) | 1.736 | [0.703, 4.287] | 0.232 |
| Mesalazine (preop. therapy) | 0.797 | [0.296, 2.145] | 0.653 |
| Enteral nutrition | 1.022 | [0.138, 7.573] | 0.983 |
| Parenteral nutrition | 1.786 | [0.402, 7.938] | 0.446 |
| Hemoglobin | 0.797 | [0.633, 0.903] | **0.046** |
| C-reactive protein | 1.180 | [0.800, 1.740] | 0.404 |
| Albumin | 1.005 | [0.755, 1.338] | 0.974 |
| Leukocytes | 1.000 | [1.000, 1.000] | 0.277 |
| Previous abdominal surgery | 2.226 | [1.624, 5.149] | **0.047** |
| Timing (elective vs urgency) | 2.738 | [0.504, 14.874] | 0.243 |
| Type of surgical access | 0.743 | [0.284, 1.944] | 0.545 |
| Conversion | 3.000 | [0.543, 16.585] | 0.208 |
| Type of anastomosis | 1.150 | [0.528, 2.503] | 0.725 |
| Technique (stapled vs handsewn) | 3.316 | [1.836, 13.158] | 0.051 |
| Postoperative drainage | 0.978 | [0.059, 16.121] | 0.988 |
| Blood transfusion | 1.682 | [0.506, 5.586] | 0.396 |
| Length of stay | 1.017 | [0.952, 1.088] | 0.613 |
| Postoperative therapy | 0.538 | [0.718, 4.081] | 0.225 |
| Timing of postoperative therapy | 1.026 | [0.268, 3.923] | 0.970 |
