# Supplementary material for: Risk Factors for Complications and Disease Recurrence after Ileocecal Resection for Crohn’s Disease in Children and Adults
Source: Biomedicines. 2024 Apr 13;12(4):862. doi: 10.3390/biomedicines12040862 (PMC11047859; doi:10.3390/biomedicines12040862)
Supplement: Supplementary file 1 [file biomedicines-12-00862-s001.zip › Table S5_new.docx]

Table S5. Results of multivariate logistic regression models for surgical disease recurrence in Crohn’s disease patients

|  |  | **Multivariate** |  |
| --- | --- | --- | --- |
| **Variable** | **OR** | **[95% CI]** | ***p*-value** |
| Group (pediatric vs adult) | 2.100 | [0.434, 10.168] | 0.357 |
| Gender (male vs female) | 0.944 | [0.289, 3.082] | 0.925 |
| Age at diagnosis | 1.099 | [0.499, 2.421] | 0.814 |
| Age at surgery | 0.997 | [0.966, 1.029] | 0.871 |
| Smoker | 3.405 | [0.884, 13.120] | 0.075 |
| Disease duration | 1.003 | [0.997, 1.009] | 0.320 |
| Disease location | 0.567 | [0.278, 1.159] | 0.120 |
| Perianal disease | 4.145 | [1.867, 19.822] | **0.045** |
| Stricturing disease | 1.857 | [0.219, 15.771] | 0.571 |
| Penetrating disease | 1.680 | [0.527, 5.352] | 0.380 |
| Steroids (preop. therapy) | 0.290 | [0.035, 2.372] | 0.248 |
| Biologics (preop. therapy) | 3.240 | [0.841, 12.488] | 0.088 |
| Methotrexate (preop. therapy) | 1.061 | [0.209, 5.393] | 0.943 |
| Thiopurines (preop. therapy) | 2.143 | [0.666, 6.900] | 0.201 |
| Mesalazine (preop. therapy) | 0.583 | [0.119, 2.849] | 0.505 |
| Enteral nutrition | 2.000 | [0.193, 20.723] | 0.561 |
| Parenteral nutrition | 4.145 | [0.867, 10.822] | 0.075 |
| Hemoglobin | 0.943 | [0.688, 1.294] | 0.718 |
| C-reactive protein | 1.566 | [0.706, 3.475] | 0.270 |
| Albumin | 1.246 | [0.571, 2.717] | 0.581 |
| Leukocytes | 1.000 | [1.000, 1.000] | 0.272 |
| Previous abdominal surgery | 1.500 | [0.480, 4.686] | 0.485 |
| Timing (urgency vs elective) | 5.250 | [1.034, 26.656] | **0.045** |
| Type of surgical access | 0.502 | [0.148, 1.696] | 0.267 |
| Conversion | 3.371 | [0.548, 20.754] | 0.190 |
| Type of anastomosis | 0.607 | [0.244, 1.511] | 0.283 |
| Technique (stapled vs handsewn) | 2.134 | [0.253, 17.988] | 0.486 |
| Blood transfusion | 1.882 | [0.446, 7.931] | 0.389 |
| Length of stay | 1.056 | [0.975, 1.143] | 0.179 |
| Postoperative therapy | 0.851 | [0.261, 2.770] | 0.788 |
| Timing of postoperative therapy | 0.365 | [0.037, 3.652] | 0.391 |
